# Application of Mini-CEX combined with DOPS in standardized training of community outpatient residents

**DOI:** 10.1186/s12909-024-05739-x

**Published:** 2024-07-19

**Authors:** Yuanmei Wang, Gang Chen, Jie Chen, Fan Jiang

**Affiliations:** Department of General Practice, Wuhan Fourth Hospital, Qiaokou District, 493 Hanzheng Street, Wuhan, Hubei 430030 China

**Keywords:** Resident, Standardization, Mini-CEX, DOPS, General outpatient, Community

## Abstract

**Objective:**

To explore the application effect of mini clinical evaluation exercise (Mini-CEX) combined with direct observation of procedural skills (DOPS) in the standardized training of general practitioners in community clinics.

**Methods:**

From June 2022 to June 2023,20 general practitioners who received standardized training for residents in the general outpatient department of Changqing Community Health Service Center of Wuhan Fourth Hospital were collected as the research objects. Mini-CEX combined with DOPS was used to evaluate the general practitioners at the time of admission, 2 weeks of training and 4 weeks of training, and the results were fed back.

**Results:**

The scores of 20 general practitioners at 2 weeks and 4 weeks of training were compared with the scores at the time of admission, and the difference was statistically significant, *p* < 0.05.

**Conclusion:**

Mini-CEX combined with DOPS can improve the teaching effect of standardized training of residents in community general clinics.

**Supplementary Information:**

The online version contains supplementary material available at 10.1186/s12909-024-05739-x.

## Background

At present, 80% of medical consumption in China is completed in tertiary hospitals, and 80% of medical expenses are spent on the middle and later stages of chronic diseases. Therefore, the Chinese government has proposed a strong grassroots medical strategy. The development of general practice is a strategic decision of the country, and the key to promoting basic medical care is high-quality general practitioners [1]. The nature of general practice determines that community outpatient medical and health services are the main part of general practitioners, and it is advisable to strengthen the training of community general outpatient clinics. According to the survey, outpatient teaching often lacks teacher-student interaction and communication, or general practitioners do not actively participate in the clinical diagnosis and treatment process, the assessment mechanism is single, and the assessment content is out of touch with the general practice work [2–4].General practitioners are difficult to lay a theoretical and practical foundation for independent consultation in the general practice clinic in the future. In view of the above shortcomings of outpatient teaching, this study actively explores a new teaching mode of standardized training of general practitioners in line with China’s reality and international standards. Actively exploring teaching methods that can improve the clinical abilities of general practitioners. In recent years, formative assessment methods such as mini clinical evaluation exercise ( Mini-CEX) and direct observation of procedural skills ( DOPS) have been gradually introduced into China. Formative evaluation has the characteristics of timely feedback. In the teaching process, it can effectively improve students’ learning ability and teachers’ teaching level by promoting and improving evaluation. It has been adopted by more and more educators [5–9]. This study intends to use Mini-CEX combined with DOPS to evaluate the clinical reception process and practical skills operation process of general practitioners in community general practice clinics,evaluate the clinical abilities of general practitioners,and to give timely evaluation and feedback, aiming to strengthen the training effect of general practitioners in community clinics.

## Methods

### Study participants

The research subjects were selected as 20 general practitioners who underwent standardized training for resident physicians at the General Clinic of Changqing Community Health Service Center of Wuhan Fourth Hospital from June 2022 to June 2023. The average age was 25 years old, with 6 males and 14 females. The project was approved by the Ethics committee of Wuhan Fourth Hospital. Informed consent was given by all participants. All residents consented to participate in the study.

### Study methods

Mini CEX is used for evaluating the diagnosis and treatment ability of general practitioners in community general practice clinics, while DOPS is used for evaluating the operational skills of community general practice clinics. The Mini CEX form includes 7 aspects: medical history collection, physical examination, humanistic care, clinical judgment, health education consultation, organizational effectiveness, and overall performance (see Caption 1 for details).

The DOPS form includes 9 aspects: knowing indications and contraindications, informing patients and obtaining consent, being familiar with operational preparation, having a good aseptic concept, correct and standardized operating steps, accurate and proficient techniques, seeking assistance, post operative treatment, and overall performance. Each project adopts a 9-point system, with scores of 1–3 points for non-compliance, 4–6 points for compliance, and 7–9 points for outstanding performance (see Caption 2 for details).

### Evaluation outcomes

The evaluation indicators will include a Mini CEX and DOPS evaluation of 20 students at the time of enrollment, 2 weeks of training, and 4 weeks of training, comparing the differences between the previous and subsequent three evaluations. Design a questionnaire survey, which includes: mobilizing learning interest; Improve the ability to think, ask questions, and solve problems; Cultivate clinical thinking; Developing self-expression skills; Increase clinical learning confidence; Increase the learning burden; Students are not nervous during the assessment; Suitable for general discipline training. Complete after the evaluation is completed. The answer options use the Likert 5-level scoring system, which includes strongly agree, agree, neutral, disagree, and strongly disagree (see Caption 3 for details).

### Questionnaire survey

The questionnaire is distributed by a teacher arranged by the project team, who is responsible for collecting and checking whether the questionnaire is complete and clear. It is filled out by 20 students, and each student is responsible for filling out one questionnaire. A total of 20 questionnaires are distributed, and 20 valid questionnaires are collected, with an effective recovery rate of 100%.

### Quality control

Teachers enthusiastic about outpatient practice and teaching were selected for Mini-CEX and DOPS training to ensure consistency.

### Statistical analysis

The SPSS 30.0 statistical software was used for statistical analysis of data. Quantitative data were expressed by $\left(\overline{\text{x}}\pm \text{s}\right)$ for repeated measurement data analysis of variance over three time periods. *P* < 0.05 indicates a statistically significant difference.

## Results

### Comparison of Mini-CEX scores at different time periods

The results showed that the score of Mini CEX was significantly improved at 2 and 4 weeks of training compared to the time of enrollment, with a statistically significant difference of *p* < 0.05 (see Table 1 for details).

**Table 1:** The Mini CEX scores of 20 general practice residents were compared at different time periods

Project	Enrollment	At 2 weeks	At 4 weeks	95%CI	*p*
Consultation skills	2.80 ± 0.69	3.70 ± 0.57	6.10 ± 0.91	4.01–4.39	< 0.001
Physical examination	2.25 ± 0.55	2.90 ± 0.55	5.35 ± 0.87	3.33–3.68	< 0.001
Clinical judgment ability	2.80 ± 0.61	3.85 ± 0.49	5.70 ± 1.08	3.92–4.32	< 0.001
Humanistic concern	2.25 ± 0.55	2.80 ± 0.77	4.95 ± 0.83	3.15–3.52	< 0.001
Professional consulting ability	2.20 ± 0.41	2.40 ± 0.60	4.40 ± 0.68	2.85–3.15	< 0.001
Organizational skills	2.60 ± 0.60	3.60 ± 0.50	5.10 ± 1.12	3.56–3.97	< 0.001
Organizing ability	2.55 ± 0.51	3.70 ± 0.57	5.45 ± 1.42	3.70–4.10	< 0.001

*Mini CEX* mini clinical exercise evaluation

### Comparison of DOPS scores at different time periods

The DOPS scores of 20 general practice residents were compared at different time periods. The results showed that the scores of resident physicians significantly improved at 2 and 4 weeks of training compared to when they entered the department, with a statistically significant difference of *p* < 0.05 (see Table 2).

**Table 2:** The DOPS scores of 20 general practice residents were compared at different time periods

Project	Enrollment	At 2 weeks	At 4 weeks	95%CI	*p*
Indications and contraindications	2.55 ± 0.61	3.65 ± 0.49	6.10 ± 0.91	3.92–4.28	< 0.001
Inform patients	2.10 ± 0.31	2.90 ± 0.64	5.35 ± 0.86	3.28–3.62	< 0.001
Familiarize	2.30 ± 0.47	3.60 ± 0.88	5.70 ± 1.08	3.65–4.09	< 0.001
Aseptic concept	2.35 ± 0.49	3.55 ± 0.61	4.95 ± 0.83	3.45–3.79	< 0.001
Operating specifications	2.50 ± 0.51	3.65 ± 0.67	4.40 ± 0.68	3.36–3.68	< 0.001
Proficient	2.40 ± 0.50	3.75 ± 0.72	5.10 ± 1.11	3.54–3.96	< 0.001
Overall performance	2.50 ± 0.51	4.05 ± 0.76	5.45 ± 1.10	3.79–4.21	< 0.001
Seek help	2.50 ± 0.51	4.05 ± 0.95	4.50 ± 0.61	3.50–3.87	< 0.001
Post operation processing	2.50 ± 0.51	3.80 ± 0.69	4.80 ± 0.89	3.51–3.89	< 0.001

*DOPS* direct observation and evaluation of operational skills

### Students’ feedback on Mini-CEX and DOPS evaluation

Feedback from students on the Mini CEX and DOPS teaching mode: A questionnaire survey shows that 15% (3/20) of students believe that adding Mini CEX and DOPS to teaching increases the learning burden, but 85% of students recognize and support the positive effect of adding Mini CEX and DOPS to outpatient teaching evaluation mode. The specific situation is shown in Table 3.

**Table 3:** Feedback of resident residents on Mini-CEX and DOPS evaluation mode

Item	Number of people who agree	Percent (%)
Stimulates learning interest	19	95.0
Improves thinking and problem-solving skills	19	95.0
Trains clinical thinking	18	90.0
Trains self-expression skills	18	90.0
Improves clinical learning confidence	17	85.0
Increases learning burden	3	15.0
Residents are not nervous during examination	15	75.0
Suitable for standardized training in general practice	17	85.0

*Mini-CEX* mini-clinical evaluation exercise, *DOPS* direct observation and evaluation of operational skills

## Discussion

Mini-CEX is an assessment tool developed and recommended by the American Board of Internal Medicine (ABIM) on the basis of Mini-CEX in 1995 to evaluate the clinical ability of residents and has teaching function [10]. DOPS evaluation was first designed by the Royal College of Physicians (RCP) in 2007 [11]. Teachers directly observe students performing clinical technical operations on real patients and give immediate feedback. Both Mini-CEX and DOPS are carried out in a real clinical environment for real patients. They meet the requirements of post competency and are suitable for evaluating the actual performance of residents in the work, which is conducive to training and learning [12, 13].

In this study, both Mini-CEX and DOPS can let the teacher understand the resident’s basic knowledge reserve, basic operation, basic skills, clinical diagnosis and treatment thinking ability and the ability to solve practical problems, and emphasize the weak links of the resident in the training. Learning is conducive to the resident’s reflection and adjustment of the problems encountered in the training, and improves the enthusiasm and urgency of the resident’s learning, emphasis has been placed on the cultivation of clinical abilities. This study shows that various indicators of Mini CEX and DOPS have been significantly improved, and the clinical diagnostic and therapeutic thinking ability of resident physicians has made significant progress. The application of this new teaching model has enhanced the clinical ability of general practitioners.

At present, the ability and clinical skills of general practitioners need to be improved [14]. After standardized training in internal medicine, surgery, gynecology, pediatrics and other departments, general practitioners have qualified to grow into a general practitioner, but not a general practitioner in the true sense. In this study, in the real working environment of the community general practice clinic, the general practitioner completed the transition and transformation of the professional environment and work role, and cultivated the grass-roots practical ability of the general practitioner.

At this stage, there are some problems in the grass-roots practice base [15], heavy public health light basic medical ability training, less access opportunities, teaching outpatients for chronic patients [16], did not get the grass-roots diagnosis and treatment practice ability training, training can not meet the needs of use, training and use of the phenomenon of segmentation is widespread [17]. This study aims to improve and explore how to utilize limited teaching practice to address the problems existing in grassroots practice bases, so that general practitioners can quickly and effectively grasp the diagnosis and treatment thinking of common and frequently occurring diseases in their profession and possess certain clinical abilities.

Different scholars have conducted a series of studies on the teaching processes, content, and forms involved in medical teaching activities, constantly exploring more optimized teaching methods [18–23]. Chen Haoyang [24] suggested that on the basis of the traditional assessment mode, Mini-CEX and DOPS should be introduced to strengthen the assessment and evaluation of clinical ability in the evaluation of the current situation of the standardized training of residents in China. Chen Xiaolei et al. [25] found that in the teaching of general practice, the introduction of network teaching platform and situational participatory teaching mode, and the use of formative evaluation methods have improved students’ interest and enthusiasm in learning. Yao Yundie [5–9, 26] used formative assessment in cardiology, general practice and other departments, which is conducive to the reform and improvement of clinical training and education training mode, and the satisfaction of residents with teaching is also significantly improved. In this study, the introduction of Mini-CEX and DOPS in community outpatient teaching has increased the learning interest and enthusiasm of general practitioners, strengthened the cultivation of clinical abilities, and achieved certain results.

The standardized training of residents is different from the internship stage. After the training, the general practitioner will work in the community health service center, and must be able to complete the routine diagnosis and treatment activities independently. In introducing the general practice training system in the United Kingdom, Yao Mi et al. [27] proposed that attention should be paid to workplace assessment and teacher-student interaction feedback. This study focuses on the community general practice clinic, and trains residents to be familiar with and master the routine diagnosis and treatment and operation of community general practice clinic diseases. A number of studies have shown that the demand-driven and workplace-based integrated assessment courses Mini-CEX and DOPS can not only improve the overall satisfaction of students, but also improve the quality of teaching in an effective and resource-saving way [28–30].

In summary, Mini-CEX and DOPS can bring benefits to the teaching and evaluation of resident training, and benefits for the improvement of clinical abilities of general practitioners and have been recognized by them. It is worthy of further development and continuous improvement in the community general practice clinic. There are still some shortcomings in this study, and there is no in-depth analysis combined with test scores, and not combined with specific cases. The evaluation of Mini-CEX and DOPS is also subjective, and it is planned to carry out relevant research in the next step.

### Supplementary Information

Additional file 1. Mini-CEX test.

Additional file 2. DOPS.

Additional file 3. Categorization of evaluators’ feedback in feedback analysis.

Additional file 4.

## Data Availability

Data are available from the corresponding author on reasonable request.
