# Supplementary material for: Application of Mini-CEX combined with DOPS in standardized training of community outpatient residents
Source: BMC Med Educ. 2024 Jul 19;24:780. doi: 10.1186/s12909-024-05739-x (PMC11264813; doi:10.1186/s12909-024-05739-x)
Supplement: Supplementary file 1 — Additional file 1. Mini-CEX test. [file 12909_2024_5739_MOESM1_ESM.docx]

**Caption 1 Mini clinical drill (Mini-CEX) rating scale**

Assessment time: year moon Nikkobe:

Student name: □ Pearson □ Intern □ Continuing sher

Teacher: □ associate chief physician □ attending physician □ chief resident physician □ resident physician □ high year graduate student

Location: □ outpatient □ emergency □ ward □ □ office □ treatment room □ operating room

Patient: □ Male □ Female age: □ Outpatients □ inpatients □ standardized patients

diagnose: Complexity: □ low □ high in □

Exercise and evaluation focus: □ Medical history inquiry □ physical examination □ clinical judgment □ humanistic care

□ Methodist consultation □ organizational effectiveness □ overall performance

| **Assessment project** | **difference** | **same as** | **outstanding** |
| --- | --- | --- | --- |
| 1. Medical history inquiry | □ Call the patient □ self-introduction □ can encourage the patient to describe the medical history □ appropriate questions and guidance to obtain correct and sufficient information □ to have an appropriate response to the patient's emotional and body language | | |
|  | □1 □2 □3 | □4 □5 □6 | □7 □8 □9 |
| 2. Physical examination | □ Inform the patient of the purpose and scope of the examination □ pay attention to the secrecy of the examination site □ Conduct a comprehensive and focused examination according to the condition □ Correct operation and implementation steps □ appropriately and carefully handle the discomfort of the patient's | | |
|  | □1 □2 □3 | □4 □5 □6 | □7 □8 □9 |
| 3. Clinical judgment | □ Ability to summarize medical history and physical examination data □ ability to interpret relevant examination results □ Ability of differential diagnosis □ rationality and logic of judgment □ can judge the benefits, risks and costs of treatment | | |
|  | □1 □2 □3 | □4 □5 □6 | □7 □8 □9 |
| 4. Humanistic care | □ Show respect and care □ establish good relationship and interdependence □ concern and deal with patient discomfort □ respect patient privacy □ appropriately meet the needs of patients to seek relevant information | | |
|  | □1 □2 □3 | □4 □5 □6 | □7 □8 □9 |
| 5. Health advice | □ Explain examination and treatment □ Explain examination results and clinical relevance □ related treatment and counseling | | |
|  | □1 □2 □3 | □4 □5 □6 | □7 □8 □9 |
| 6. Organizational efficiency | □ Can handle in a reasonable order □ timely and appropriate □ experience and indirect | | |
|  | □1 □2 □3 | □4 □5 □6 | □7 □8 □9 |
| 7. Overall performance | □1 □2 □3 | □4 □5 □6 | □7 □8 □9 |

Direct observation time: Minutes of feedback time: minute

Participation criteria: not standard (1-2); close (3-4); basic standard (5-6); complete standard (7-8)

Student satisfaction with this assessment: low □ 1 □ 2 □ 3 | □ 4 □ 5 □ 6 | □ 7 □ 8 □ 9 high

Teacher comments:

Teacher's signature:
