# Supplementary material for: Application of Mini-CEX combined with DOPS in standardized training of community outpatient residents
Source: BMC Med Educ. 2024 Jul 19;24:780. doi: 10.1186/s12909-024-05739-x (PMC11264813; doi:10.1186/s12909-024-05739-x)
Supplement: Supplementary file 3 — Additional file 3. Categorization of evaluators’ feedback in feedback analysis. [file 12909_2024_5739_MOESM3_ESM.docx]

Caption 3 Feedback and evaluation of the students

1. Mobilize interest in learning:

1) very agree 2) agree 3) neutral 4) oppose 5) very opposed

2. Improve the ability of thinking, asking questions and solving problems:

1) very agree 2) agree 3) neutral 4) oppose 5) very opposed

3. Cultivate clinical thinking:

1) very agree 2) agree 3) neutral 4) oppose 5) very opposed

4. Cultivate self-expression ability:

1) very agree 2) agree 3) neutral 4) oppose 5) very opposed

5. Increase the confidence in clinical learning:

1) very agree 2) agree 3) neutral 4) oppose 5) very opposed

6. Increase the learning burden:

1) very agree 2) agree 3) neutral 4) oppose 5) very opposed

7. Students are not nervous in the assessment:

1) very agree 2) agree 3) neutral 4) oppose 5) very opposed

8. Suitable for general practice and training:

1) very agree 2) agree 3) neutral 4) oppose 5) very opposed
